# Comprehensive Study of the Current-Induced Spin–Orbit Torque Perpendicular Effective Field in Asymmetric Multilayers

**DOI:** 10.3390/nano12111887

**Published:** 2022-05-31

**Authors:** Baoshan Cui, Zengtai Zhu, Chuangwen Wu, Xiaobin Guo, Zhuyang Nie, Hao Wu, Tengyu Guo, Peng Chen, Dongfeng Zheng, Tian Yu, Li Xi, Zhongming Zeng, Shiheng Liang, Guangyu Zhang, Guoqiang Yu, Kang L. Wang

**Affiliations:** 1 Songshan Lake Materials Laboratory, Dongguan 523808, China; cuibaoshan@sslab.org.cn (B.C.); zhuzengtai@sslab.org.cn (Z.Z.); 202111105010070@stu.hubu.edu.cn (C.W.); guotengyu@sslab.org.cn (T.G.); chenpeng@sslab.org.cn (P.C.); zhengdongfeng@sslab.org.cn (D.Z.); gyzhang@iphy.ac.cn (G.Z.); 2 Beijing National Laboratory for Condensed Matter Physics, Institute of Physics, Chinese Academy of Sciences, Beijing 100190, China; 2017141531037@stu.scu.edu.cn; 3 Key Laboratory for Magnetism and Magnetic Materials of Ministry of Education, School of Physical Science and Technology, Lanzhou University, Lanzhou 730000, China; xili@lzu.edu.cn; 4 Faculty of Physics and Electronic Science, Hubei University, Wuhan 430062, China; shihengliang@hubu.edu.cn; 5 School of Physics & Optoelectric Engineering, Guangdong University of Technology, Guangzhou 510006, China; guoxb@gdut.edu.cn; 6 College of Physics, Sichuan University, Chengdu 610064, China; work_tian@scu.edu.cn; 7 Department of Electrical Engineering, University of California, Los Angeles, CA 90095, USA; wang@ee.ucla.edu; 8 Nanofabrication Facility, Suzhou Institute of Nano-Tech and Nano-Bionics, Chinese Academy of Sciences, Suzhou 215123, China; zmzeng2012@sinano.ac.cn

**Keywords:** spin–orbit torque, perpendicular magnetic anisotropy, perpendicular effective field, zero-field switching

## Abstract

The spin–orbit torques (SOTs) in the heavy metal (HM)/ferromagnetic metal (FM) structure hold promise for next-generation low-power and high-density spintronic memory and logic applications. For the SOT switching of a perpendicular magnetization, an external magnetic field is inevitable for breaking the mirror symmetry, which is not practical for high-density nanoelectronics applications. In this work, we study the current-induced field-free SOT switching and SOT perpendicular effective field ($H_{z}^{eff}$) in a variety of laterally asymmetric multilayers, where the asymmetry is introduced by growing the FM layer in a wedge shape. We show that the design of structural asymmetry by wedging the FM layer is a universal scheme for realizing field-free SOT switching. Moreover, by comparing the FM layer thickness dependence of ($H_{z}^{eff}$) in different samples, we show that the efficiency (*β =* $H_{z}^{eff}$/*J*, *J* is the current density) is sensitive to the HM/FM interface and the FM layer thickness. The sign of *β* for thin FM thicknesses is related to the spin Hall angle (*θ*_SH_) of the HM layer attached to the FM layer. *β* changes its sign with the thickness of the FM layer increasing, which may be caused by the thickness dependence of the work function of FM. These results show the possibility of engineering the deterministic field-free switching by combining the symmetry breaking and the materials design of the HM/FM interface.

Current-induced spin–orbit torque (SOT) provides an energy-efficient and fast way to electrically manipulate the magnetization [1,2,3,4] and dynamics of spin textures (such as chiral domain wall (DW) [5,6,7,8,9] and magnetic skyrmions [10,11,12], etc.) in the heavy metal (HM)/ferromagnetic metal (FM) multilayers. In such a structure, an in-plane current (*I*) flowing through the HM layer is converted to a pure spin current (*J*_s_) due to the spin Hall effect [1,13,14] and/or interfacial Rashba effect [15]. The *J*_s_ injects into the adjacent FM layer and thus exerts the SOTs. To enable the SOT-driven perpendicular magnetization switching, an external magnetic field is inevitable to break the mirror symmetry [2], which is impractical for high-density nanoelectronics applications. Until 2014, the field-free SOT switching of a perpendicular magnetization was achieved by introducing a laterally asymmetric structure [16], providing a new pathway to realize all-electric deterministic switching. After that, many other strategies have been proposed for realizing field-free SOT switching [17,18,19,20,21,22,23,24,25,26,27,28,29,30,31]. For the case of laterally asymmetric structure, the field-free SOT switching is driven by the current-induced out-of-plane effective magnetic field ($H_{z}^{eff}$). The magnitude and sign of $H_{z}^{eff}$ determine the switching efficiency and switching polarity at zero external field, respectively. However, the key factors that affect the magnitude and sign of $H_{z}^{eff}$ are still elusive.

In this work, we aim to explore the key factors that affect the current-induced $H_{z}^{eff}$ and the resulting field-free SOT switching in a variety of laterally asymmetric structures. We find that the $H_{z}^{eff}$ is generally introduced in various laterally asymmetric structures. By comparing the FM thickness dependence of the efficiency (*β*) of $H_{z}^{eff}$ (i.e., *β =* $H_{z}^{eff}$/*J*, where *J* is the current density), we show that *β* is closely related to the HM/FM interface and the FM layer thickness. Our results advance the understanding of the current-induced out-of-plane effective magnetic field in the laterally asymmetric structures.

The film stacks consisting of (i) Ta(5)/Gd(1)/CoFeB(*w*)/MgO(2), (ii) Pt(5)/CoFeB(*w*)/MgO(2), (iii) IrMn(5)/CoFeB(*w*)/MgO(2), (iv) Ta(5)/CoFeB(*w*)/MgO(2), (v) Ta(5)/Mo(1)/CoFeB(*w*)/MgO(2), and (vi) W(5)/CoFeB(*w*)/MgO(2) (thickness in nm) were prepared by magnetron sputtering at room temperature on Si substrates capped with a 100 nm thermal oxide under a base pressure of <1 × 10^−8^ Torr. The CoFeB layer was grown by the oblique sputtering method and hence has a wedge-sharp structure (*w*). The CoFeB layer thickness (denoted as *t*_CoFeB_) varies from 0.50 nm to 1.20 nm within the lateral length of ~5 cm. It is worth noting that we calibrate the wedged thickness in a large lateral scale, therefore, the several nm-scale thickness difference can be detected precisely. The other layers were uniformly grown by rotating the substrate during the deposition. The stacks were annealed at 250 °C for 30 min to enhance the perpendicular magnetic anisotropy (PMA). The basic magnetic properties of the different samples are similar, therefore, only the results of the Ta(5)/Gd(1)/CoFeB(w)/MgO(2) multilayer are presented. The schematic illustration of the Ta/Gd/CoFeB/MgO structure is shown in Figure 1a. The films were patterned into Hall bar devices with the dimension of 130 × 20 μm^2^ (see Figure 1b) via standard photolithography and dry etching techniques for anomalous Hall effect (AHE) and magneto-optical Kerr effect (MOKE) microscopy measurements. For the Hall bar device, there could be some thickness variation, however, the wedged trend should be kept.

Figure 1c shows the AHE loops of the devices with a series of *t*_CoFeB_, in which the *R*_H_ and *H*_z_ are the Hall resistance and out-of-plane external magnetic field, respectively. The sharp-square loops indicate the existence of a PMA for the devices. The dynamics of the domain wall driven by *H*_z_ for the whole Hall bar device with *t*_CoFeB_ = 0.70 nm is shown in Figure 1d. In image ①, the red dotted line shows the current channel of the Hall bar device. At first, a large *H*_z_ along +*z* direction was applied to saturate the sample, and the picture was chosen as the reference as shown in image ①. As *H*_z_ increases in the −*z* direction and reaches the switching field, a reversed domain is nucleated at the bottom edge of the device (see image ②). As the field increases, the domain expands to the whole Hall bar device, as shown in images ③–⑤. These results show that the switching is accomplished by domain nucleation and the domain wall motion. We also measured the perpendicular anisotropy energy density *K*_u_ (*K*_u_ = *μ*_0_*H*_k_*M*_s_/2). *M*_s_ is the saturation magnetization, which is measured by the superconducting quantum interference device (SQUID) and has a magnitude of ~710 emu/cm^3^, *μ*_0_ is the vacuum permeability. *H*_k_ is the effective anisotropy field, which is measured by the in-plane AHE loops, as shown in Figure 1e. It is known that *R*_H_ is only proportional to the *z*-axis component of magnetization (***M***) in a system with PMA. As the in-plane magnetic field (*H*_x_) increases, ***M*** will be rotated from the *z* direction (easy axis) to the *x* direction (hard axis). Consequently, there is a reduction of the *R*_H_ at high fields, as shown in Figure 1e. Figure 1f summarizes the *t*_CoFeB_ dependence of *K*_u_. *K*_u_ increases first when *t*_CoFeB_ < 0.77 nm, which has been attributed to the change of the CoFeB/MgO interface (i.e., the interfacial anisotropy) caused by B diffusion [32]. With further increasing the CoFeB thickness, *K*_u_ starts to decrease since the PMA has an interfacial origin [33].

Next, we show the field-free SOT switching in the devices with different *t*_CoFeB_ performed by the Keithley 2612A source/measure unit. In the measurements, the writing pulses with a width of 1 ms were injected along the device channel. To avoid applying a zero-writing current, the step number was set as 101 for scanning the writing pulse between −35 (−30) mA and 35 (30) mA. After each writing pulse, the Hall resistance was measured by a reading current of 3 mA. Figure 2a shows the field-free SOT switching for devices of 0.66 nm < *t*_CoFeB_ < 0.91 nm. The current density (*J*_e_) is calculated by assuming a uniform current distribution across the film stack. As a reference, we have measured the magnetization switching curve in the absence of external magnetic fields loop in the structure without the wedge structure, as shown in Figure 2a. One can see that there is no deterministic SOT-induced magnetization switching. The previous work has demonstrated that the field-free SOT switching was driven by the current-induced $H_{z}^{eff}$ that originates from the lateral asymmetry [16]. In detail, the wedged layer is deposited at an oblique angle with respect to the substrate surface (along the *y* axis) without rotating the substrate, so it is grown in a tilted direction away from the substrate normal. Consequently, it breaks the mirror symmetry with respect to the *x–z* plane and allows for the creation of a built-in effective electric field (***E***) along the *y* axis. Consequently, a current induced $H_{z}^{eff}$ is expected due to the Rashba spin–orbit coupling (SOC), which is expressed by $H_{z}^{eff}$= α ⸱ (***p*** × ***E***) [15,34]. Here, *α* is the Rashba SOC constant depends on the materials, and ***p*** represents the electron’s momentum. If $H_{z}^{eff}$ is larger than the coercivity, the magnetization switching can be achieved, although the thickness gradient is very tiny. Figure 2b shows the SOT switching loops under different *H*_x_ for the device with *t*_CoFeB_ = 0.81 nm. We found that the switching polarity changes from a clockwise mode to an anticlockwise mode when the field is increased to *H*_x_ = 50 Oe. In this case, the switching is not dominated by the $H_{z}^{eff}$ anymore, and the conventional damping-like SOT dominates the switching with the assistance of the in-plane magnetic field. It is worth noting that the switching polarity under a large positive *H*_x_ is consistent with the case in the Ta/CoFeB/MgO system [2]. Nevertheless, the Gd has a positive spin Hall angle (*θ*_SH_) [35], which is opposite to Ta. In this regard, we conclude that the conventional damping-like SOT originates from the spin current that is generated in the Ta layer and diffuses through the Gd layer even it is partially compensated by the spin current in the Gd layer.

During the current-driven magnetization switching, the MOKE measurements were performed simultaneously. Figure 2c shows the MOKE images of SOT switching by using the same Hall bar device as shown in Figure 1d. During the measurements, a large *H*_z_ along +*z* direction was first applied to saturate the magnetization, and the picture was chosen as the reference, as shown in image ①. Then, the current pulses were applied to drive the magnetization switching. Interestingly, the nucleation position is different from that in Figure 1d. For the SOT-driven switching, the initial reversal of domain occurs on the right side of the device channel, as shown in image ②, which is likely due to the presence of Dzyaloshinskii–Moriya interaction (DMI) [36,37] that tilts the magnetization at the device boundary [38], lowering the SOT switching barrier for the right edge and thus to induce the domain nucleation. As the current increases, the DW is subsequently driven to the left side of the device channel. For the observed DW, the neighboring magnetizations on its two sides point ↑↓. When a current is applied along +*x* axis, the spin orientation (***σ***) of the net spin current is along +*y* direction. The dimpling-like field (***H*_DL_**) as a driven force of the DW can be expressed as ***H*_DL_** = ***m*** **× *σ***, pointing respectively along +*z* or −*z* direction when the magnetic moment (***m***) in the DW along +*x* (→) or −*x* (←) directions. Here, “→” and “←” refer to the in-plane component of ***m*** in the center of DW. Obviously, the current along +*x* drives the DW to move along −*x* direction, namely, an effective ***H*_DL_**along −*z* direction is generated. Thus, the magnetic configuration in the domain wall is ↑←↓, i.e., a left-handed chirality. The magnitude of DMI can be obtained by measuring the AHE loops of switching a Hall cross with combined *H*_x_ and *H*_z_ under a series of DC current densities [39,40]. Figure 2d summarizes the SOT efficiency (*χ*) as a function of *H*_x_. The saturated field with the maximum *χ* corresponds to the effective DMI field (|*H*_DMI_|). Then, the DMI exchange constant (|*D*|) can be obtained by using |*D*| = *μ*_0_*M*_s_*Δ*|*H*_DMI_| [41], where Δ is the DW width and is related to exchange stiffness constant *A* ≈ 1.5 × 10^−11^ J/m and *K*_u_, with the form of Δ = (*A*/*K*_u_)^1/2^ [6,39]. The value of the |*D*| scales linearly with the inverse of *t*_CoFeB_, as shown in Figure 2e, indicating its interfacial origin.

In the following, we extract the current-induced $H_{z}^{eff}$. Figure 3a shows the hysteresis AHE loops under currents with opposite polarities. The AHE loop shifts to the left under a negative current, indicating the existence of a perpendicular effective field along +*z* direction ($H_{z}^{+}$). Similarly, a positive current generates an effective perpendicular field along the −*z* direction ($H_{z}^{-}$). The averaged perpendicular effective field $H_{z}^{eff}$ can be obtained by $H_{z}^{eff}$ = ($H_{z}^{-}$ − $H_{z}^{+}$)/2 = *βJ*, where *β* and *J* refer to the efficiency of $H_{z}^{eff}$ and the current density, respectively. Figure 3b shows the *t*_CoFeB_ dependence of *β*, where *β* decreases firstly and changes its sign at *t*_CoFeB_ ≈ 0.66 nm, after that *β* increases negatively. The sign change of *β* is consistent with our previous work [16]. It is worth noting that the *β* at 0.63 nm < *t*_CoFeB_ < 0.66 nm shows small magnitudes, which are likely responsible for the partial switching and same polarity of switching, as shown in Figure 2a.

To explore the key factors that affect the *β*, samples ii-vi were measured. Figure 4 summarizes the CoFeB thickness dependence of *β* for these samples. We found that $H_{z}^{eff}$ exists in all laterally asymmetric structures, indicating this is a universal phenomenon. The Pt/CoFeB, IrMn/CoFeB, and Gd/CoFeB samples have a similar thickness dependence, while Ta/CoFeB, Mo/CoFeB, and W/CoFeB samples show an opposite dependence. To better compare these samples, the *β*, *θ*_SH_, and *D*_s_ = *D*·*t*_FM_ are extracted for all the samples, as shown in Table 1. We note that some parameters are obtained from the literatures. We found that the interfacial HM layer attached to the FM has a significant relation to the *β*. First, the sign of *β* for the thin CoFeB region is determined by the sign of *θ*_SH_ of the interfacial HM layer attached to the FM layer. The *β* is positive (negative) for the interfacial HM that has a positive (negative) *θ*_SH_. For example, the *β* values are positive when the interfacial HM are Pt, IrMn, and Gd, which have a positive *θ*_SH_. Similarly, *β* values are negative when the interfacial HM are Ta, Mo, and W, which have a negative *θ*_SH_. Consequently, *β* is negative for Ta/CoFeB, Mo/CoFeB, and W/CoFeB samples. As the thickness of CoFeB increases, *β* changes its sign at thick CoFeB side except for the Pt/CoFeB and IrMn/CoFeB samples. Our previous work pointed out that the sign of *β* likely depends on the work functions of interfacial HM and FM [34]. Thus, the sign-change of *β* in our systems may be ascribed to the thickness dependence of work function of CoFeB layer. The sign-change was not observed for Pt and IrMn samples, which may be attributed to the fact that the PMA regions in these two systems are narrow and the sign reversal thickness is not reached. For all the samples, the magnitude of *β* strongly depends on the CoFeB thickness, which may be caused by the thickness dependences of the work function of FM layer, *K*_u_, interfacial DMI, and the oblique deposition induced crystal structure asymmetry of CoFeB layer. Further work is required to elucidate the microscopic origin of the thickness dependence.

In conclusion, we have demonstrated that the current-induced SOT perpendicular effective field is universal for a variety of laterally asymmetric multilayers with a wedged FM layer. The efficiency *β* is sensitive to the HM/FM interface and the FM layer thickness. The sign of *β* in a laterally asymmetric structure at thin FM thickness position is determined by the sign of the *θ*_SH_ of interfacial HM layer attached to the FM layer. As the thickness of FM increases, the sign reversal of *β* is observed, which may be related to the thickness dependence of the work function of FM. Our work advances the understanding of the out-of-plane effective field in the laterally asymmetric device and provides a pathway in engineering the perpendicular effective field. However, additional advantages may be added to the field-free SOT devices. For example, Pt usually introduces a large DMI [3], IrMn provides antiferromagnetic coupling [20,44], Mo improves the sample’s thermal stability [49], and W has a larger *θ*_SH_ [48].

## Figures and Tables

**Figure 1:**
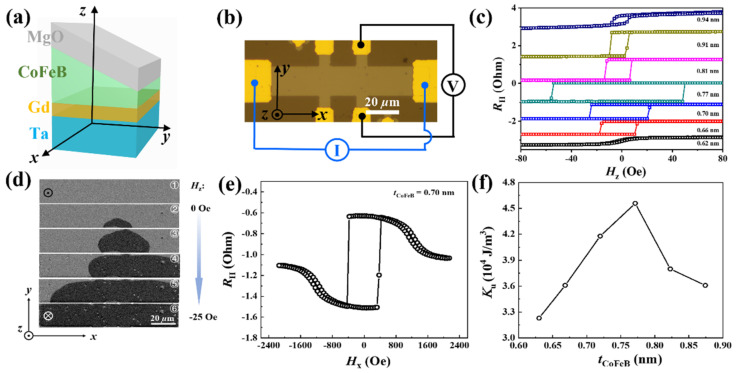
(**a**) Sketch of the multilayer stack of Ta(5)/Gd(1)/CoFeB(*w*)/MgO(2) (layer thickness in nm). (**b**) Hall bar device and the measurement configuration. (**c**) AHE loops for the devices with different CoFeB thicknesses under a current of *I* = 1 mA. (**d**) Representative MOKE images of the perpendicular magnetic field induced domain wall motion for the whole Hall bar device with *t*_CoFeB_ = 0.70 nm. (**e**) Hall resistance (*R*_H_) as a function of the in-plane magnetic field (*H*_x_). (**f**) The CoFeB thickness dependence of perpendicular magnetic anisotropy energy (*K*_u_).

**Figure 2:**
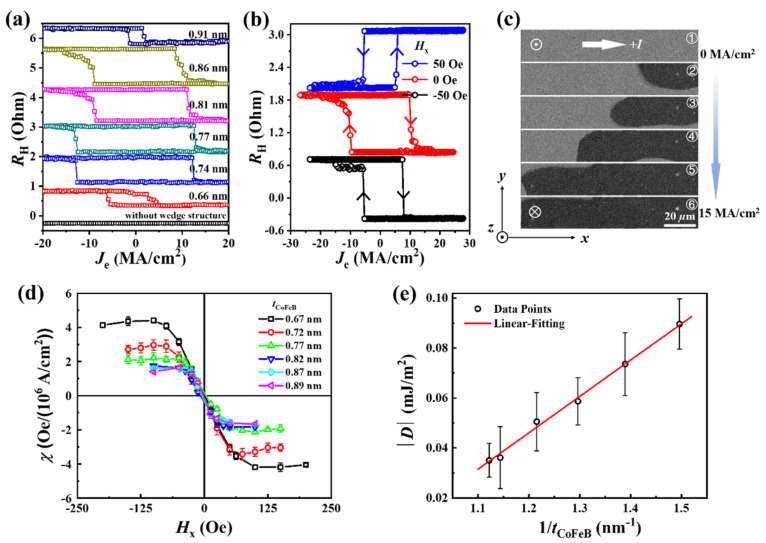
(**a**) Field-free SOT switching loops for the devices with different *t*_CoFeB_. (**b**) SOT switching at zero field and in-plane magnetic fields of *H*_x_ = ±50 Oe for the device with *t*_CoFeB_ = 0.81 nm. (**c**) Representative MOKE images of pulsed current−driven magnetization switching for the whole Hall bar device with *t*_CoFeB_ = 0.70 nm. (**d**) The measured SOT efficiency *χ* as a function of the in-plane magnetic field for different *t*_CoFeB_. (**e**) The relationship between the estimated DMI constant |*D*| and 1/*t*_CoFeB_.

**Figure 3:**
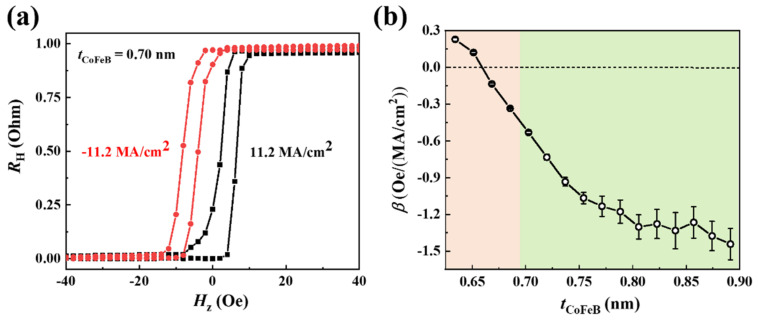
Current-induced out-of-plane effective magnetic fields measured using loops-shift methods. (**a**) Out-of-plane hysteresis loops under opposite current polarities for *t*_CoFeB_ = 0.70 nm. (**b**) *β* as a function of CoFeB thickness, where the full field-free SOT switching only can be found in the green region.

**Figure 4:**
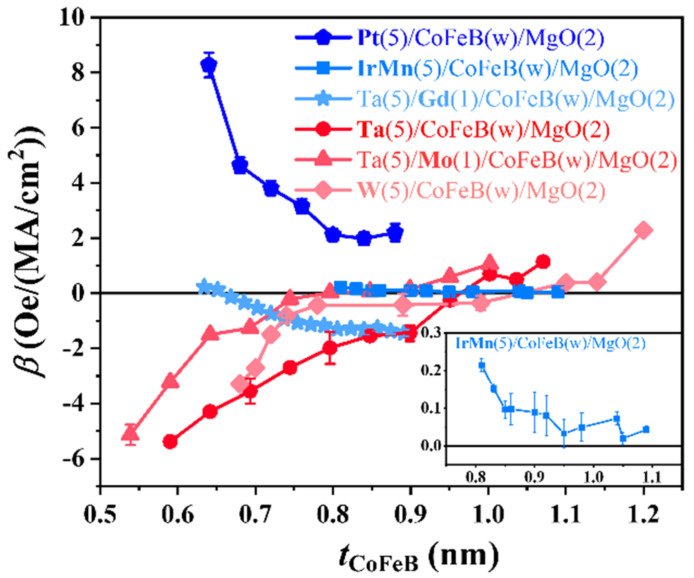
The summarized *β* as a function of wedged CoFeB thickness in X/CoFeB systems, where X are Pt, IrMn, Gd, Ta, Mo, and W. The inset shows the enlarged IrMn/CoFeB case.

**Table 1:** Room temperature *β*, *θ*_SH_, and *D*_s_ in this work.

HM	*β* (Oe/(10^6^ A/cm^2^))		*θ*_SH_	*D*_s_ (10^−15^ J/m)
	Thin CoFeB	Thick CoFeB		
Pt	8.3	2.2	0.05~0.15 [42]	−965 [43]
IrMn	0.2	0	0.057 [44]	−172 [45]
Gd	0.24	−1.44	0.04 [35]	−146
Ta	−5.5	1.2	−0.05~−0.35 [2,46]	36 [43]
Mo	−5.1	1.1	−0.003 [47]	490 [47]
W	−3.5	2.5	−0.14~−0.49 [48]	73 [43]

## Data Availability

All the data present in this paper will be made available upon reasonable request. Please contact the corresponding author for further information.
